# GDNF overexpression in astrocytes enhances branching and partially preserves hippocampal function in an Alzheimer’s rat model

**DOI:** 10.1038/s41598-025-02881-4

**Published:** 2025-06-02

**Authors:** Ana Abril Vidal Escobedo, Facundo Peralta, Gustavo Ramón Morel, Martino Avallone, Tomas Björklund, Paula Cecilia Reggiani, Joaquín Pardo

**Affiliations:** 1 Facultad de Ciencias Médicas, Instituto de Investigaciones Bioquímicas de La Plata “Profesor Doctor Rodolfo R. Brenner”, Universidad Nacional de La Plata, La Plata, Buenos Aires Argentina; 2 Molecular Neuromodulation, Wallenberg Neuroscience Center, Lund University, Lund, Sweden; 3 Cátedra de Citología, Histología y Embriología, Facultad de Ciencias Médicas, Universidad Nacional de La Plata, La Plata, Buenos Aires Argentina

**Keywords:** Astrocytes, GDNF, Hippocampus, AAV9, Neurodegeneration, Cognitive neuroscience, Glial biology, Neurotrophic factors

## Abstract

Astrocytes are essential for maintaining neuronal health and regulating the brain’s inflammatory environment. In this study, we developed an adeno-associated viral vector (AAV9) designed to selectively overexpress glial cell line-derived neurotrophic factor (GDNF) in astrocytes, using the astrocyte-specific GFAP promoter and TdTomato for transduction tracking. This approach yielded targeted GDNF expression in hippocampal astrocytes. Sholl analysis revealed that GDNF overexpression significantly enhanced astrocytic branching complexity and process length. Using the intracerebroventricular streptozotocin (STZ) model of neurodegeneration, we evaluated the impact of GDNF on astrocytic morphology, neuroinflammation, and hippocampal-dependent memory. Although GDNF prevented astrocytic process length reduction, it did not mitigate neuroinflammation, as evidenced by persistent microglial activation, nor did it improve deficits in the novel object recognition task. However, GDNF + STZ treated animals performed similarly as SHAM controls at exploring the goal sector at the Barnes Maze. These findings demonstrate the capacity of the AAV-GFAP-GDNF-TdTom construct to induce astrocytic branching and partially preserve memory function. They also underscore its partial therapeutic potential in a neuroinflammatory, metabolically compromised and neurodegenerative context.

**Supplementary Information:**

The online version contains supplementary material available at 10.1038/s41598-025-02881-4.

## Introduction

Astrocytes are essential regulators of neuronal activity, neuroinflammation, and overall brain homeostasis. As the most abundant glial cell population, they maintain dynamic interactions with neurons and the brain’s immune system, positioning them as key players in neurological and neurodegenerative conditions. Astrocyte atrophy, characterized by a reduction in their territorial domains, has been associated with early cognitive impairments due to diminished metabolic support and disrupted synaptic function^1^. This decline may also result in reduced synaptic coverage, allowing neurotransmitters to diffuse beyond synaptic clefts and contributing to neuronal hyperexcitability—a hallmark of neurodegenerative diseases^2^.

The intracerebroventricular streptozotocin (ICV-STZ) model of neurodegeneration, widely used to replicate the neuropathological characteristics of sporadic Alzheimer’s Disease (AD), induces cognitive decline, metabolic dysfunction, increased amyloidogenesis, tau pathology, oxidative stress, mitochondrial dysfunction, neuronal degeneration, synaptic loss, and chronic neuroinflammation—key hallmarks of AD^3–16^. Metabolic dysfunction induced by STZ has been linked to disrupted insulin signaling pathways, including alterations in Akt/PKB and GSK-3β activity, leading to a progressive insulin-resistant state in the brain^8,14,17^. Importantly, insulin resistance and altered glucose metabolism induced by the ICV-STZ model extends beyond neuronal dysfunction to include significant alterations in astrocyte biology. This resistance reduces glucose uptake and impairs astrocyte metabolism, exacerbating the neuroinflammatory response^8,10^. Although the ICV-STZ model does not typically produce hyperglycemia^18,19^, studies on the effects of hyperglycemia on astrocytes reveal potentially relevant mechanisms, such as increased glycolytic activity and lactate production, which can contribute to toxicity and inflammation^20^. *In vitro*, exposure of primary astrocytes to high glucose induced a metabolic shift towards glycolysis, inhibited proliferation, and caused cell cycle arrest^21^. These findings support the notion that astrocytic energy metabolism is highly sensitive to changes in glucose availability and insulin signaling, as occurs in the ICV-STZ model.

The metabolic disturbances in the ICV-STZ model lead to hippocampal astrocyte morphological changes, such as reduced process length and branching complexity, which have been associated with impaired neuroprotection and synaptic dysfunction^11,16^. Sholl analysis revealed that the first detectable change in response to STZ was a simplification of astrocytic arborization, with reduced process length, fewer main processes, and decreased branching complexity, even in the absence of overt neuronal loss^16^. These findings suggest that astrocyte structural remodeling is an early and sensitive marker of STZ-induced neurodegeneration. Notably, a reduction in astrocytic complexity has been reported in the post-mortem brains of dementia patients^22^. While the ICV-STZ model does not fully recapitulate all aspects of AD pathology, it serves as a valuable tool to study the relationship between astrocyte morphology and neuroprotection within the context of metabolic dysfunction, neurodegeneration and chronic neuroinflammation. Moreover, the time-dependent progression of neurochemical and cognitive alterations in the model—ranging from an acute response (< 1 month) to a later decompensation phase (6–9 months)—offers a window to explore therapeutic strategies for both early intervention and disease modification^12^.

In this neurodegenerative context, astrocyte-targeted approaches are particularly promising. Among neurotrophic factors implicated in brain repair, glial cell line-derived neurotrophic factor (GDNF) stands out due to its potent neuroprotective effects, particularly on dopaminergic neurons. Initially identified in the supernatant of a rat glioma cell line as a trophic factor for embryonic midbrain dopaminergic neurons, GDNF has since been extensively studied for its role in dopaminergic neuron protection in Parkinson’s Disease. These findings raised interest in GDNF-based therapies, leading to clinical trials aimed at delivering GDNF directly to the brain^23,24^. Additionally, gene therapy strategies have been explored for this purpose, including viral vectors designed to overexpress GDNF. For instance, our group previously employed a recombinant adenovirus to overexpress GDNF in the hypothalamus, which effectively reduced chronic hyperprolactinemia in senile female rats by targeting hypothalamic dopaminergic neurons^25^.

The therapeutic potential of GDNF in models of cognitive decline has been explored, although the available evidence remains limited. Mice heterozygous for a GDNF gene deletion showed poor performance in the Morris water maze, highlighting its potential involvement in spatial learning^26^. Recently, our group reported the construction of a tet-off regulatable recombinant adenovirus expressing GDNF and its successful expression in rat hippocampal cells^27^. Another promising approach involved the use of lentiviruses pseudotyped with the Mokola glycoprotein (Mokola-G) to efficiently transduce astrocytes *in vivo*. Injection of this lentivirus coding for GDNF into the hippocampus of aged Fischer 344 rats led to cognitive improvements within two weeks^28^. Further studies from the same group demonstrated that GDNF gene therapy in 3xTg-AD mice not only improved spatial learning and memory but also reduced amyloid plaque load in the hippocampus^29^. However, these studies did not reconstruct the morphology of GDNF-expressing astrocytes, leaving a gap in deciphering how GDNF influences astrocytic structure and function.

A bicistronic viral vector capable of overexpressing GDNF alongside a reporter protein in hippocampal astrocytes offers a unique opportunity to study both GDNF-mediated neuroprotection and astrocytic morphological changes. Astrocytes’ unique ability to modulate the brain microenvironment makes them an attractive target for therapeutic interventions. We recently developed an AAV9 vector system that enables the overexpression of two genes specifically in astrocytes. This vector employs the astrocyte-specific GFAP promoter (GfaABC1D)^30^ and includes endogenous TdTomato (TdTom) fluorescence for transduction tracking^31^.

Here, we report the construction of an AAV9 vector expressing both GDNF and TdTom under the GfaABC1D promoter, designed to selectively overexpress GDNF in hippocampal astrocytes. This work reports, to the best of our knowledge, the first application of such a vector in the hippocampus. To evaluate the impact of astrocytic GDNF overexpression in the context of neurodegeneration, we employed the ICV-STZ model and subjected the animals to behavioral tests involving hippocampal function. Thus, the hypothesis of this study was that GDNF overexpression in astrocytes modifies their morphology to provide protection of hippocampal-dependent cognitive function in the setting of neurodegeneration.

## Materials and methods

### Animals and ethics declaration

The experiments were performed in accordance to the Animal Welfare Guidelines and regulations of NIH (INIBIOLP’s Animal Welfare Assurance No A5647-01) and approved by the School of Medicine (National University of La Plata, Argentina) Committee for the Care and Use of Laboratory Animals (Protocol # P01-02-2021). All methods are reported in accordance with ARRIVE guidelines. Sprague Dawley rats were obtained from the School of Medicine (National University of La Plata, Argentina). For this study male rats were used. The animals were 2-months old at the beginning of the experiments, they weighed 260 ± 50 g and were housed in a temperature-controlled room (22 ± 2 ºC) on a 12:12 h light/dark cycle with food and water available ad libitum (3 animals/cage).

### Molecular cloning

DNA sequences were cloned into the pZac2.1 GfaABC1D-tdTomato AAV transfer plasmid (#44332 Addgene). The plasmid was digested with NheI and EcoRI restriction enzymes and the larger fragment was gel-extracted and subsequently used as vector backbone. The human recombinant GDNF and the IRES sequences were PCR amplified from source plasmids with compatible primers (GDNF_Fw: *ttaatacgactcactatagggccaccatgaagttatgggatgtcgtggc*, GDNF_Rv: *aggcatttcttcagatacatccacaccttttagcgg*, IRES(GDNF)_Fw: *atgtatctgaagaaatgcctagcctgcagg* and IRES_Rv: *ccttgctcaccatggtggcgttatcatcgtgtttttcaaaggaaaaccac*). The 3 DNA fragments were HIFI-assembled (#E2621 *New England Biolabs)*. The resulting AAV transfer plasmid was named GfaABC1D-GDNF-ires-tdTomato.

### AAV production

HEK293 cells were transfected with the GfaABC1D-GDNF-ires-TdTom transfer plasmid, the AAV9 capsid plasmid pAAV 2/9n (#112865 Addgene), and the pHGT-1 adenoviral helper plasmid (1.2:1:1 molar ratio). Four days after transfection AAVs were harvested using polyethylene glycol 8000 precipitation and chloroform extracted followed by PBS exchange with Amicon Ultra-0.5 Centrifugal filters (Merck Millipore). The AAVs were titrated by qPCR using the primers ITR-Fw (*GGAACCCCTAGTGATGGAGTT*) and ITR-Rv (*CGGCCTCAGTGAGCGA*). The virus batch titers were adjusted to 10^13^ genome copies per milliliter (GC/ml) for animal injection. This protocol has been thoroughly described by Negrini and cols^32^.

### Assessment of GDNF by RT-qPCR

Two rats were randomly selected and used for this experiment. They were anesthetized with a mixture of ketamine hydrochloride (90 mg/kg) and xylazine (8 mg/kg) intraperitoneally (ip) and underwent stereotaxic surgery for injection in the hippocampus using a stereotaxic apparatus (ST-51600U, Stoelting). Injections were performed using a Hamilton 701 N syringe (volume 10 µl) fitted with a 26s gauge needle (bevel tip, length 51 mm). The hippocampal coordinates relative to Bregma were: − 3.8 mm anteroposterior, 2 mm lateral, and − 3.2 mm ventral^33^. One animal was bilaterally injected in the hippocampus, whereas the second one was unilaterally injected in the left hippocampus with the AAV9-(GFAP)-GDNF-TdTom vector. The injection volume was 2 µl/side. Four weeks later, the animals were euthanized under deep isoflurane anesthesia followed by rapid decapitation and their hippocampi dissected out and snap-frozen until use. For RNA extraction, the hippocampi were homogenized in TRIzol Reagent (#15596026 Thermo Fisher Scientific), and the RNA isolated according to the manufacturer’s instructions. Subsequently, 1 µg RNA was reverse transcribed with Superscript IV Reverse Transcriptase (#18-090-050 Thermo Fisher Scientific). Afterwards, the resulting cDNA was 1:5 diluted and 1 µl of it was used as template for quantitative PCR (qPCR) reaction (Ssoadvanced™ Universal SYBR, #1725271 BioRAD) in a CFX96 touch real-time PCR detection system (Bio-Rad, Hercules, CA, USA). As a control, we used cDNA samples from hippocampi of an animal injected with a control vector where GFP replaces GDNF: AAV9-(GFAP)-GFP-TdTom^31^. Thus, there were *N* = 3 hippocampi for GFP and GDNF groups. Data were normalized against rat β-actin expression. The primers used were β-actin-Fw *(GACGTTGACATCCGTAAAGACC)*, β-actin-Rv *(CTAGGAGCCAGGGCAGTAATCT)*, GDNF-Fw *(GCGCTGAGCAGTGACTCAAA)* and GDNF-Rv *(TCTGGCCTCTCCGACCTTT)*. Relative GDNF mRNA expression was quantified using the 2^−ΔCt method, with β-actin as the reference gene. Briefly, for each sample, ΔCt was calculated as Ct_GDNF − Ct_β-actin, and GDNF expression relative to β-actin was computed as 2^−ΔCt. All values were expressed relative to the GFP-injected hippocampus with the lowest GDNF expression and log₁₀-transformed.

### Experimental design for the astrocyte branching study in Naïve animals

Four rats were randomly selected and bilaterally injected in the hippocampus as described in “Assessment of GDNF by RT-qPCR”. Two rats were injected with the control virus AAV9-(GFAP)-GFP-TdTom (GFP group, *N* = 4 hippocampi) and two rats were injected with the vector AAV9-(GFAP)-GDNF-TdTom (GDNF group, *N* = 4 hippocampi). Four weeks later, the animals were placed under deep isoflurane anesthesia and perfused with phosphate-buffered paraformaldehyde 4% (pH 7.4) fixative. The brains were removed and stored in paraformaldehyde 4% (pH 7.4) overnight (4 ºC). Afterwards, brains were maintained in cryoprotectant solution (30% ethylene glycol, 30% sucrose, in PB 0.05 M) at -20ºC until sectioning and subsequent GFAP staining (see below). The brain from one animal of the GDNF group was used to take photographs showing the expression of TdTom and GDNF.

### Experimental design for the STZ experiment

Rats were randomly divided into four groups (*n* = 8/group): SHAM, STZ, GFP + STZ, and GDNF + STZ. For experimental controls, three groups were used: SHAM, STZ, and GFP + STZ. All animals were anesthetized and underwent stereotaxic surgery for bilateral intrahippocampal injections as described in “Assessment of GDNF by RT-qPCR”. The GDNF + STZ and GFP + STZ groups received intrahippocampal injections of AAV9-(GFAP)-GDNF-TdTom and AAV9-(GFAP)-GFP-TdTom (control virus), respectively. The SHAM and STZ groups received artificial cerebrospinal fluid (aCSF: 120 mM NaCl, 3 mM KCl, 1.15 mM CaCl2, 0.8 mM MgCl2, 27 mM NaHCO3, and 0.33 mM NaH2PO4, pH 7.4) at 2 µl/side. Four weeks later, the animals were anesthetized again and underwent a second stereotaxic surgery for bilateral ICV injections using the following coordinates: − 0.9 mm anteroposterior, ± 1.5 mm lateral, and − 4.5 mm ventral, to bregma. The STZ, GFP + STZ, and GDNF + STZ groups received an ICV injection of STZ (Sigma-Aldrich, CAS#18883-66-4) at a dose of 3 mg/kg^16^, resulting in an injection of 4–6 µl, depending on animal’s weight. The SHAM group received an ICV injection of aCSF using the same procedure. Three weeks later, the animals were subjected to behavioral tests, as described below, and subsequently euthanized under deep isoflurane anesthesia followed by rapid decapitation. Left brain hemispheres were fixed in 4% paraformaldehyde in PBS overnight at 4 °C. Afterwards, the paraformaldehyde solution was replaced with a 30% sucrose-PB 0.05 M solution for 24 h. This solution was replaced with a cryoprotectant solution, and brains were kept at -20 °C until sectioning.

### Behavioral tests

#### Barnes maze

We used a modified Barnes Maze (BM) protocol previously documented^34,35^. The rats were placed in an elevated circular platform (120 cm diameter) with 20 holes at the periphery, one of which was connected to an escape box, named as hole 0, the remaining holes were numbered 1 to 10 clockwise, and − 1 to − 9 counterclockwise. On the sides of the maze there were visual cues. During the task animals received escape stimuli consisting of a 90-dB white noise generated by an MP3 audio track played through speakers connected to a computer, and a 500-W incandescent floodlight provided the light stimulus. These stimuli were manually controlled by the experimenter. In the acquisition trials (ATs), animals explored the platform for 2 min or until they found the escape box. After six repeated ATs (2 ATs/day), which is the learning stage, animals were submitted to the probe trial (PT), in which the escape box was removed. In this last trial, we assessed the goal sector exploration as the spatial reference memory of the animals. ATs and PT were recorded for subsequent offline analysis, and after each individual testing, the platform was cleaned with ethanol 10% to avoid olfactory cues. A hole exploration was considered as a rat introduced its head into a hole and passed through the plane of the table. As behavioral readouts, the following parameters were assessed:

- Latency (ATs): time (in seconds) spent by an animal from its release on the platform until it enters the escape box.
- Total explorations (PT): Number of hole explorations during the probe trial.
- Goal sector exploration (PT): Defined as the total number of explorations in holes − 1, 0, and 1.

#### Novel object recognition

The arena consisted in a square box (65 × 45 × 65 cm; W x H x D). One day following habituation, the rats were presented two identical objects referred to as familiar objects (FO) for 5 min. After 90 min, animals were introduced to the same arena for another 5-minute session, during which one of the items was replaced by a novel object (NO) with similar dimensions but a different shape and color. Following each trial, both objects and the box were cleaned with 10% ethanol. The time spent exploring each object was measured, afterwards, a discrimination index (D.I.) was calculated by taking the difference in exploration time between NO and FO and dividing this value by the total exploration time: $\:D.I.=(NOt-FOt)/(NOt+FOt)$, where t=time, a positive score indicates more time exploring the NO, a negative score indicates more time exploring the FO, and a score of zero indicates a neutral preference. Finally, the D.I. values for every group were tested against the hypothetical value D.I. = 0 by one-sample hypothesis testing^35^.

### Brain processing and immunohistochemistry

The brains were cut coronally in 40-µm-thick sections with a vibratome (Leica) at a 1 in 12 section sampling regime. The sections were stored in cryoprotectant solution at -20ºC until staining, when section sampling was used as 1/12th or 1/6th. Brain staining was performed by the free-floating method using 3,3’-Diaminobenzidine (DAB) or immunofluorescence. During all incubation steps, PBS (pH 7.4) was used as buffer, and sections were rinsed three times for 15 min in PBS between each step. For the DAB staining protocol, sections were treated with a 3% H_2_O_2_ and 10% methanol solution for 1 h to quench endogenous peroxidase activity. Blocking was performed for 1 h in a solution containing 0.25% Triton X-100 and 5% serum from the secondary antibody species. Sections were then incubated at 4 °C for 48 h with the primary antibody diluted in the blocking solution. This was followed by a 2-hour incubation with a biotinylated secondary antibody, also diluted in blocking solution, and a 1-hour incubation with Avidin-Biotin complex (RRID: AB_2336819). The signal was developed using the DAB Peroxidase Substrate Kit (RRID: AB_2336382) according to the manufacturer’s protocol. Primary antibodies used for DAB staining were: anti-mCherry (gt, 1:1000, RRID: AB_2619713), anti-GDNF (gt, 1:1000, RRID: AB_2111398), and anti-Iba1 (rb, 1:1000, RRID: AB_839504). The biotinylated secondary antibodies were anti-gt (1:200, Vector Laboratories RRID: AB_2336123) and anti-rb (1:200, RRID: AB_2313606).

For immunofluorescence, sections were blocked in a solution containing 0.25% Triton X-100 and 5% donkey serum for 1 h. Then, they were incubated with primary antibodies diluted in blocking solution for 48 h at 4 °C. Afterwards, sections were incubated for 2 h with Alexa-Fluor-conjugated secondary antibody diluted in blocking solution. Finally, nuclei were stained with DAPI for 5 min. For GFAP immunofluorescence the primary antibody used was anti-GFAP (rb, 1:1000, RRID: AB_10013382) and the secondary antibody was AF 647 anti-Rb (dk, 1:500, RRID: AB_2536183).

### Microglial cell analysis

To estimate microglial cell density, brain sections from a 1-in-6 sampling interval were analyzed. Three 60X magnification images were captured in the stratum radiatum (SR) of hippocampal sections. These fields were located below the CA1 pyramidal cell layer: The central field was positioned at the injection site; since the needle tract was not easily observed—particularly in animals injected with aCSF—its location was estimated based on stereotaxic coordinates. In addition, two other fields—one to the left and one to the right of the central point—were included to ensure representative sampling. The distance between adjacent fields was 285 μm, center to center, determined by the width of the image captured at 60X magnification. Composite images were created to encompass all visible cells within each field. A counting frame of 5.2 × 10^4^ μm² was overlaid, and cells were manually counted. Iba1-positive cells were manually classified as non-reactive or reactive based on established morphological criteria^36,37^. Briefly, the non-reactive category included cells with less than or equal to five branches or cells with more than five processes and small somas. Reactive microglia were defined as cells displaying enlarged somas with retracted and thickened processes, or an amoeboid morphology characterized by numerous processes and intense Iba1 immunostaining. The density of non-reactive and reactive microglia was then calculated by dividing the cell counts by the sampled volume. Iba1R% was defined as the percentage of reactive microglia relative to the total microglia: Iba1R% = (Density of reactive Iba1-positive cells / Density of total Iba1-positive cells) × 100. Additionally, Iba1 cells immunoreactive area was assessed as follows: the above-mentioned images of Iba1 stained sections were segmented using the histogram-based model in Image Pro Plus v5.1 software (RRID: SCR_007369). This allowed the automated identification of individual microglial cells within the region of interest. The software output a list of detected cell areas, from which the mean Iba1 + immunoreactive cellular area was computed for every animal.

### Astrocyte analysis

Astrocytic branching complexity was assessed using Sholl analysis. Hippocampal sections from a 1-in-12 sampling interval were processed for GFAP immunofluorescence and imaged by confocal microscopy. Z-stack images were acquired at 63X magnification at the hippocampal SR. Twenty astrocytes per animal were selected from maximum intensity projection images. Inclusion criteria required that each cell was entirely within the field of view and clearly distinguishable from neighboring cells. TdTom fluorescence did not reliably reveal the full extent of astrocyte morphology. In many cases, the cytoplasmic signal lacked sufficient resolution to clearly distinguish distal processes. In the GFP, GDNF, GFP + STZ, and GDNF + STZ groups, the TdTom signal was used to identify transduced cells, and morphological reconstructions were performed using the GFAP channel. In the SHAM and STZ groups, GFAP-positive astrocytes were selected and analyzed accordingly. This approach allowed for direct comparison of astrocyte morphology across groups, regardless of viral transduction status.

Sholl analysis was performed in ImageJ using the Sholl Analysis plugin (http://ghoshlab.org/software/), with concentric circles spaced 5 μm apart from the soma center. The number of intersections per radius was counted for each cell. Total astrocyte process length was estimated by summing the number of intersections across all radii and multiplying by 5 μm. For each animal, the mean branching profile (intersections per radius) and mean process length were calculated from the individual astrocyte values and used as the unit of analysis in group comparisons.

### Bright-Field microscopy

Bright-field images were obtained using an Olympus BX61VS microscope equipped with Olympus VS-ASW 2.9 software and with an Olympus BX-51 microscope attached to an Olympus DP70 CCD camera driven by Image Pro-Plus v5.1 software (RRID: SCR_007369).

###  Laser scanning confocal microscopy

Confocal images were captured using a Leica SP8 laser scanning microscope. All images were acquired with a HyD detector, and laser channels were activated sequentially to prevent cross-excitation. Solid-state lasers operating at 405, 448, 552, and 650 nm excited their respective fluorophores. Imaging parameters included a pinhole set to 1 Airy Unit and a 1024 × 1024 pixels resolution. A Leica 63X/1.40 oil-immersion objective was used for image acquisition.

### Data analysis and statistics

Astrocyte analysis, behavior data analysis, and microglial quantification were done by a researcher blinded to the rat group. All statistical analyses and plots were produced using the SPSS software (version 29, IBM, Armonk, NY, USA, RRID: SCR_002865). Data were tested for normality using the Shapiro-Wilk test and for homogeneity of variances using Levene’s test. Parametric tests were used for data meeting the assumptions of normality and homogeneity of variances. When variances were not homogeneous, nonparametric alternatives were employed. An independent samples t-test was conducted to compare GDNF mRNA expression levels between GFP and GDNF groups. For astrocyte complexity analysis, a general linear model (GLM) with repeated measures was used to examine the number of intersections at increasing distances (10–50 μm) from the soma. The within-subject factor was radius, and the between-subject factor was group. Pairwise group comparisons were Bonferroni-adjusted. Astrocyte length was compared between GFP and GDNF groups using an independent samples t-test. In the BM, for the acquisition phase, a repeated measures GLM was used to analyze latency across the six trials among the experimental groups. For the NOR test, one-sample t-tests were used to compare the discrimination index (DI) against the value of 0 for each group (SHAM, STZ, STZ + GFP, and STZ + GDNF) to assess significant preference for the novel object.

## Results

### A bicistronic AAV9 vector overexpressing GDNF and TdTom increases the process length of rat hippocampal astrocytes

**Fig. 1:**
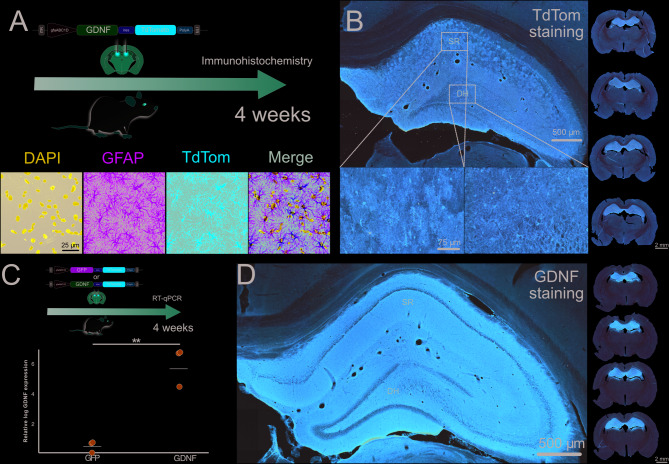
Hippocampal expression of the AAV9-(GFAP)-GDNF-TdTom vector. **(A)** Schematic of the experimental design to assess the expression of the vector and immunofluorescence images showing colocalization of TdTom in GFAP + astrocytes. **(B)** Left: pictures of a hippocampal coronal section DAB-stained for TdTom from an AAV injected animal. *Scale bar at the left inset applies to the right one.* Right: low magnification pictures of coronal brain sections DAB-stained for TdTom in animals injected in the hippocampus with the AAV9-(GFAP)-GDNF-TdTom. **(C)** Schematic of the experimental design for the qPCR study to assess GDNF levels in injected animals and the corresponding mean-annotated scatter plot. Data are referred to the GFP sample with lowest detection. *N* = 3 hippocampi/group. ** *p* < 0.01. **(D)** Left: picture of a hippocampal coronal section DAB-stained for GDNF from an AAV9-(GFAP)-GDNF-TdTom injected animal. Right: low magnification pictures of coronal brain sections DAB-stained for GDNF in the animal injected in the hippocampus with the AAV9-(GFAP)-GDNF-TdTom. *SR*: *Stratum Radiatum. DH: Dentate Hilus*.

We constructed an AAV9 coding for GDNF, the independent ribosome entry site (ires), and the red fluorescent protein TdTom under the control of the astrocyte specific promoter GfaABC1D. This vector was named AAV9-(GFAP)-GDNF-TdTom. In a previous work we had observed that this system expresses transgenes thoroughly at 4 weeks post-injection^31^. In a rat bilaterally injected in the hippocampus we observed by means of double immunofluorescence that astrocytes expressed red fluorescence in GFAP + cells (Fig. 1A). Additionally, with TdTom DAB staining we observed that the vector transduced astrocytes along the hippocampal rostrocaudal-axis (Fig. 1B). To assess the levels of GDNF expression, we injected animals in the hippocampus, extracted RNA from it, and performed RT-qPCR with primers targeting GDNF. We compared the results against animals injected with a control vector where GFP replaces GDNF: AAV9-(GFAP)-GFP-TdTom. GDNF expression was significantly higher in the GDNF group (independent samples t-test, t (4) = -6.97, *p* = 0.001) (Fig. 1C). We performed DAB staining with a GDNF antibody on sections of a rat bilaterally injected with the AAV9-(GFAP)-GDNF-TdTom vector and observed strong immunoreactivity throughout the hippocampus (Fig. 1D). To test staining specificity, we also stained a coronal brain section from an animal injected in the right hippocampus with the AAV9-(GFAP)-GFP-TdTom vector and observed no detectable signal (Suppl. Figure 1). Although no formal quantification was performed due to the overt difference in staining, this qualitative analysis revealed a marked contrast between conditions and supports robust GDNF protein expression from the AAV9-(GFAP)-GDNF-TdTom vector.

**Fig. 2:**
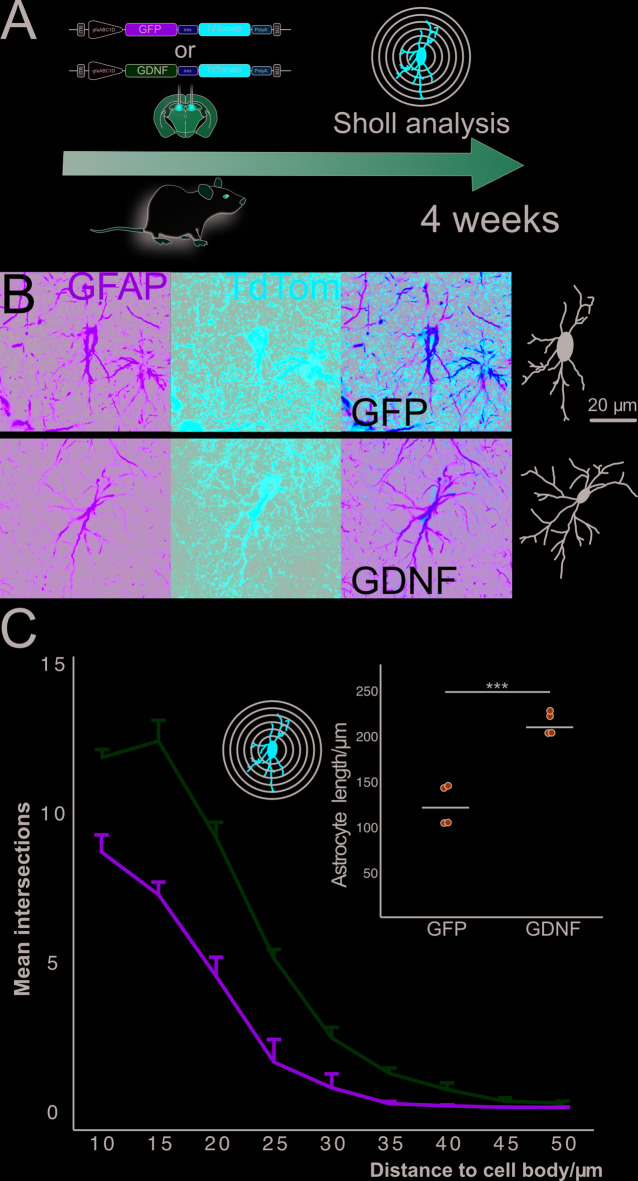
Increased branching in GDNF + astrocytes. **(A)** Schematic for the experimental design to study astrocyte branching by Sholl analysis. **(B)** Representative pictures from the GFP and GDNF groups showing astrocyte reconstruction from confocal images. The scale bar applies to all images. **(C)** Main plot: line plot showing mean astrocyte intersections at different radii for the GFP and GDNF groups. Error bars represent 1 SEM. Inset: mean-annotated scatter plot showing astrocyte length. *N* = 4 hippocampi/group. ***p < 0.001.

We posited that GDNF overexpression would increase astrocyte length. To test this hypothesis, we injected rats with AAV9-(GFAP)-GDNF-TdTom and the control virus AAV9-(GFAP)-GFP-TdTom. Four weeks later, transduced astrocytes were identified based on red fluorescence (TdTom+) and their morphology was manually reconstructed (Fig. 2A-B). A Sholl analysis was performed to study astrocyte complexity by measuring the number of intersections at different distances (10 μm to 50 μm) from the soma. A GLM with repeated measures was used to evaluate the effect of group (GFP vs. GDNF) and distance (radius) on the number of intersections. A significant main effect of radius was detected (F(8,48) = 312.986, *p* < 0.001), indicating that the number of intersections varied significantly across distances from the cell body. The group × radius interaction was also significant (F(8,48) = 13.191, *p* < 0.001), demonstrating that the pattern of intersections across radii differed between the GFP and GDNF groups. Interestingly, a significant main effect of group was observed (F(1,6) = 46.552, *p* < 0.001), with astrocytes in the GDNF group exhibiting a significantly higher average number of intersections compared to the GFP group. Notably, the number of intersections in the GDNF group was consistently higher at radii closer to the soma (10–30 μm), with the differences diminishing at larger distances. Furthermore, cells from the GDNF group displayed significantly higher process length than the GFP control (independent samples t t-test, t (6) = -6.89, *p* < 0.001) (Fig. 2C). Hence, the AAV9-(GFAP)-GDNF-TdTom vector overexpresses GDNF and TdTom in hipppocampal astrocytes at 4 weeks after injection and this prompts a significant increase in astrocyte process length.

### Astrocytic GDNF partially prevents memory deficit in the STZ neurodegeneration model

**Fig. 3:**
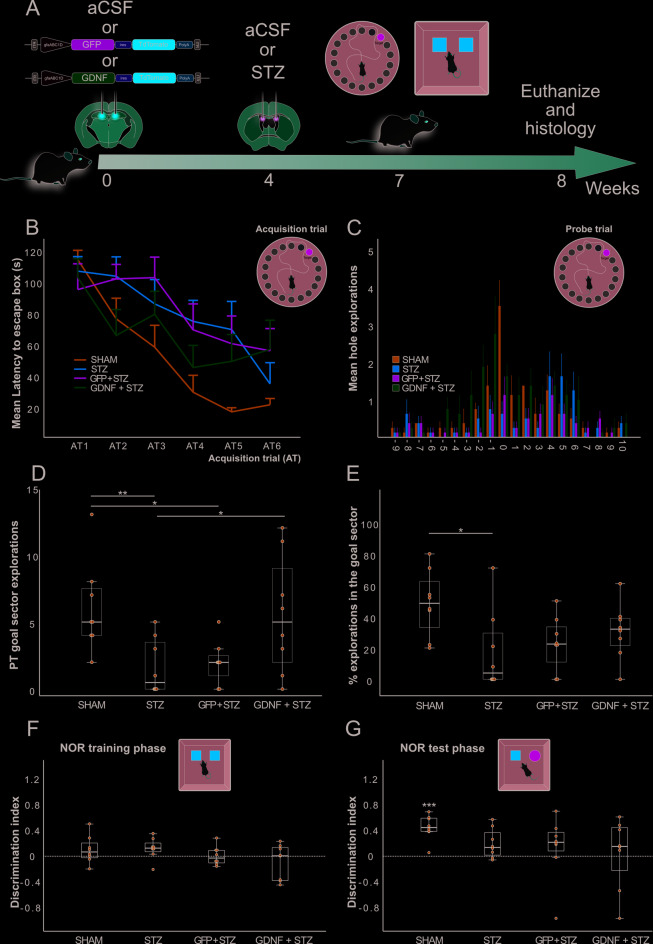
Behavior at the STZ neurodegeneration model. **(A)** Schematic for the design of the STZ study. The behavioral test pictures represent the BM and NOR tests. **(B)** Line plot showing group means displaying the latency for the BM ATs. Error bars represent 1 SEM. **(C)** Bar plot showing the mean goal exploration for the experimental groups. Error bars represent 1 SEM. **(D)** Box plot with overlaid scatter plot showing explorations at the goal sector in the BM PT. **(E)** Box plot with overlaid scatter plot showing the percentage of explorations at the goal sector. *p < 0.05, **p < 0.01. **(F)** Box plot with overlaid scatter plot showing the NOR D.I. during the training phase. **(G)** Box plot with overlaid scatter plot showing the NOR D.I. during the test phase. *** one-sample t-test against D.I.=0, *p* < 0.001.

The STZ toxin prompts neuroinflammation and cognitive deficit upon ICV injection. We hypothesized that GDNF + astrocytes with increased process length would protect the animals against STZ toxicity. To test this, 4 weeks after AAV injection, we challenged the rats with ICV STZ. Three weeks later the animals were tested in the BM and the NOR paradigms. We used SHAM, STZ and GFP + STZ animals as control groups (Fig. 3A). In the BM, a GLM with repeated measures was conducted to examine latency differences across the six trials (AT1–AT6) among the four experimental groups (SHAM, STZ, GFP + STZ, GDNF + STZ). A significant main effect of trial was observed (F(5,140) = 22.295, *p* < 0.001), indicating that latency decreased across trials, consistent with learning in the maze. A significant interaction between trial and group was detected (F(15,140) = 1.820, *p* = 0.037), suggesting that the rate of improvement in latency varied between groups. Post hoc analyses using Tukey’s HSD, however, revealed no significant pairwise differences between groups (*p* > 0.05). The main effect of group on overall latency was not significant (F(3,28) = 1.783), *p* = 0.173), indicating no significant differences in mean latencies across groups when averaged over all trials. However, the SHAM group consistently had the lowest latencies after the AT3 (Fig. 3B). In the PT, SHAM animals largely explored the goal sector (Fig. 3C). Even though no differences were recorded in total explorations (one way ANOVA F(3,28) = 2.29, *p* = 0.10) (Suppl Fig. 2), there were differences among the groups at preference for the goal sector (Kruskal Wallis H(3) = 10.98, *p* = 0.012). Bonferroni-corrected pairwise comparisons revealed that SHAM animals explored this area to a greater extent than STZ and GFP + STZ groups (*p* = 0.0060 and *p* = 0.014, respectively). There were no statistically significant differences between SHAM and GDNF + STZ (*p* = 0.52) nor between STZ and GFP + STZ groups (*p* = 0.78). Interestingly, GDNF + STZ animals outperformed their STZ and GFP + STZ counterparts, but the difference in goal sector exploration for the latter was not statistically significant (*p* = 0.034 and *p* = 0.071, respectively) (Fig. 3D). We also calculated goal sector exploration as percentage of total explorations (percentage of explorations in the goal sector). This measurement differed significantly among groups (one-way ANOVA: F(3,28) = 3.46, *p* = 0.030). Post hoc analysis showed that SHAM animals outperformed the STZ and the GFP + STZ animals, albeit the difference was statistically significant only for the former (*p* = 0.026 and *p* = 0.087, respectively). Notably, SHAM and GDNF + STZ animals showed a statistically similar percentage of explorations in the goal sector (*p* = 0.33). Even though not statistically significant, the GDNF + STZ group displayed a higher percentage of explorations in the goal sector than the STZ and GFP + STZ groups (Fig. 3E). As this assessment is influenced by overall exploration, the interpretation must be made in the context of motivation and search behavior. Overall, BM data suggest that astrocytic GDNF partially prevented spatial memory impairment in this neurodegeneration model.

The animals were also tested in the NOR paradigm. Whereas in the training phase all groups did not show a D.I. significantly higher than 0, during the test phase only the SHAM group showed a D.I. significantly greater than 0 (One sample Student’s t test against D.I. = 0, t(7) = 6.50, *p* < 0.001) (Fig. 3F-G). Hence, GDNF + STZ animals exhibited partial preservation of cognitive function, as evidenced by improved performance in some but not all behavioral measures, within the context of STZ-induced neurodegeneration.

### GDNF AAV9 vector prevented STZ-mediated astrocyte process length reduction in the hippocampus

**Fig. 4:**
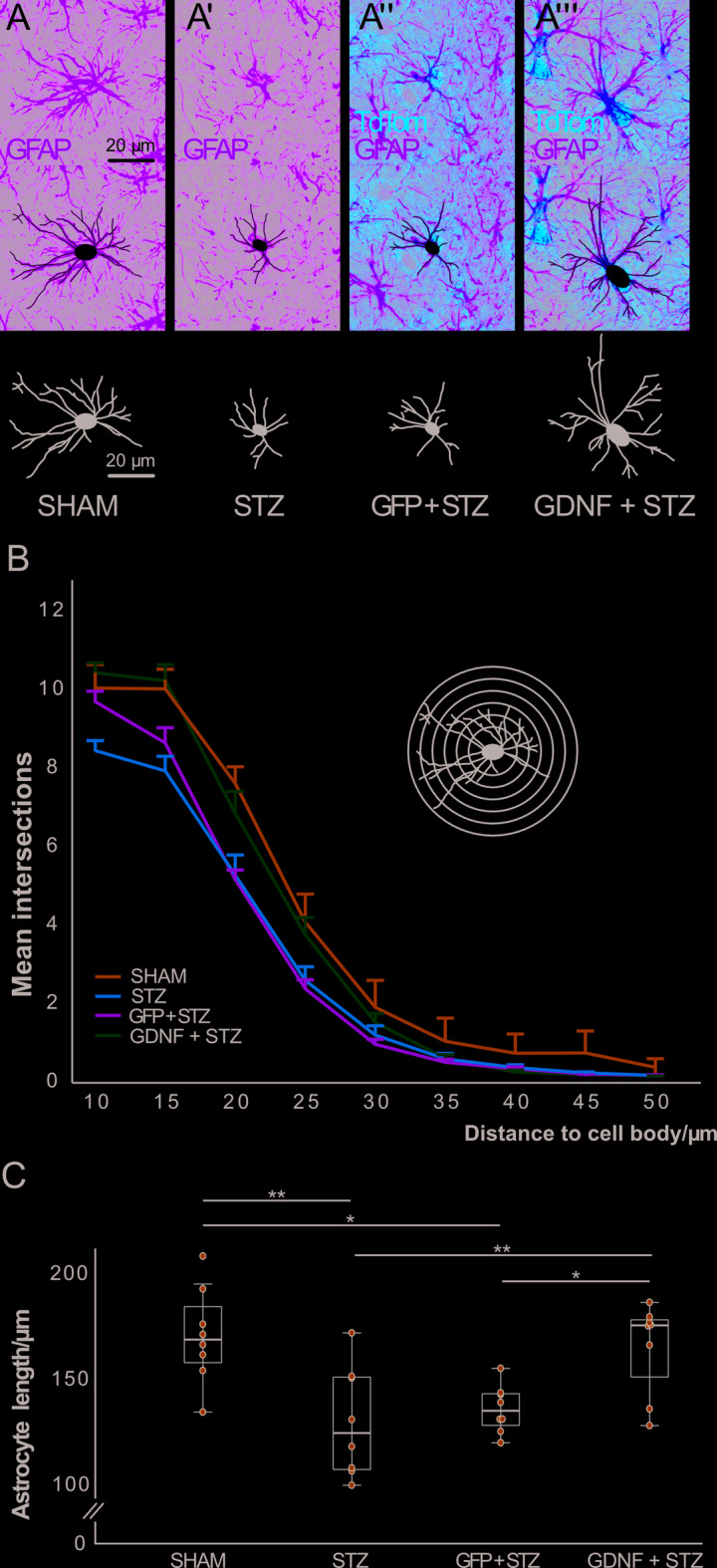
Microscopic analysis of the hippocampal astrocytes in the STZ experiment. **(A)** High magnification image of a representative astrocyte stained for GFAP from the SHAM group and cell reconstruction. **(A’)** Image of a representative cell from the STZ group. **(A’’)** Representative image of an astrocyte from the GFP + STZ group. The cell was identified with TdTom fluorescence and then reconstructed based on the morphology observed in the GFAP channel. **(A’’’)** A representative image of an astrocyte from the GDNF + STZ group. *Scale in* **(A)** *applies to* **(A-A’’’)**. **(B)** Line plot showing group means displaying astrocyte intersections at different radii. *Error bars represent 1 SEM*. **(C)** Box plot with overlaid scatter plot showing the mean astrocyte process length for the animal groups. *N* = 8/group. *p < 0.05, **p < 0.01.

Hippocampal astrocytes are susceptible to process length reduction upon STZ toxicity. Based on the experiment in naïve animals (Fig. 2), we posited that GDNF + STZ astrocytes would display increased process length than astrocytes belonging to the STZ and GFP + STZ control groups. To test this, we submitted reconstructed astrocytes from the four groups to Sholl analysis. In the case of the AAV groups, only those TdTom + were considered (Fig. 4A). A GLM with repeated measures was conducted to assess the number of intersections at increasing distances (10 μm to 50 μm) from the soma among the four groups: SHAM, STZ, GFP + STZ, and GDNF + STZ. A significant main effect of radius was detected (F(8,224) = 709.28), *p* < 0.001), indicating that the number of intersections decreased significantly with increasing distance from the soma. The radius × group interaction was also significant (F(24,224) = 3.000, *p* < 0.001), showing that the pattern of intersections across radii differed among the groups. A significant main effect of group was observed (F(3,28) = 5.290, *p* = 0.005). Pairwise comparisons (Bonferroni-corrected) revealed that SHAM animals had significantly more intersections compared to STZ (*p* = 0.011) and GFP + STZ (*p* = 0.030). However, no significant difference was found between SHAM and GDNF + STZ (*p* = 0.81) (Fig. 4B). Also, astrocyte lengths were estimated and a one-way ANOVA was performed to compare the values among the four groups. A significant main effect of group was found (F(3,28) = 7.755, *p* < 0.001). Tukey’s HSD post hoc test revealed that SHAM animals had astrocytes with increased process length compared to STZ and GFP + STZ animals (*p* = 0.003 and *p* = 0.016, respectively). GDNF-treated astrocytes also displayed significantly increased process length than those in the STZ and GFP + STZ groups (*p* = 0.008 and *p* = 0.035, respectively). Importantly, no significant difference was observed between SHAM and GDNF + STZ groups (*p* = 0.99) (Fig. 4C). Hence, GDNF prevented process length reduction in hippocampal astrocytes treated with STZ.

### Increased neuroinflammation in the hippocampus of STZ-injected animals

**Fig. 5:**
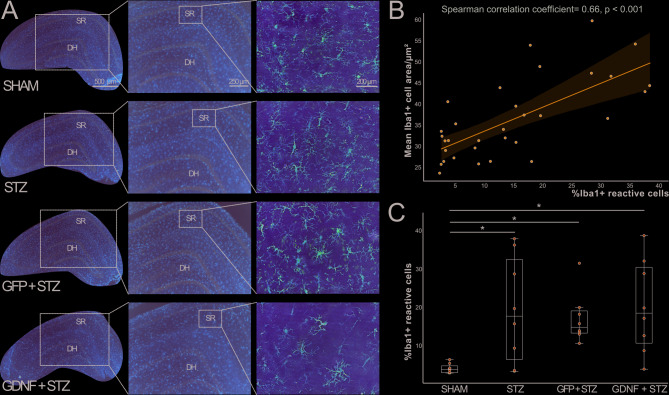
Iba1+ microglial cells quantification in the hippocampus. **(A)** Representative images showing the hippocampus of brain coronal sections DAB-stained for Iba1. *Scale bars apply to every picture of the same magnification.* **(B)** Line plot with superimposed scatter plot illustrating the correlation between Iba1 + mean cell area and the percentage of reactive microglia for the animals of the STZ study. **(C)** Box plot with overlaid scatter plot displaying the percentage of reactive microglia in the hippocampal *SR*. *N* = 8/group. *p < 0.05. *SR*: *Stratum Radiatum. DH: Dentate Hilus*.

In previous studies, we observed that Insulin-Like Growth Factor 1 (IGF1) ameliorated neuroinflammation and improved memory in the STZ model^31,35^. Thus, neuroinflammation alleviation would be relevant to improve memory deficit in this model. We stained for Iba1 and compared GDNF + STZ animals with the SHAM, STZ, and GFP + STZ controls (Fig. 5A). A one-way ANOVA was conducted to compare the total number of Iba1-positive cells across the four experimental groups. No significant effect of group was observed (F(3,28) = 1.936, *p* = 0.147), indicating that the total number of Iba1-positive cells did not differ significantly among the groups. Afterwards, we classified microglial cells as non-reactive or reactive. Based on the classification criteria, we hypothesized that the mean immunoreactive area of the cells would correlate to the percentage of reactive microglia for every animal. In this line, we observed a significant correlation between these measurements (Spearman correlation coefficient 0.66, *p* < 0.001) (Fig. 5B).

A Kruskal-Wallis test was conducted to compare the percentage of reactive microglia across the four groups. The test revealed a significant effect of group (H(3) = 12.918, *p* = 0.005). Bonferroni-corrected pairwise comparisons indicated that the SHAM group displayed a significantly fewer percentage of Iba1 + reactive cells compared to STZ, GFP + STZ, and GDNF + STZ animals (*p* = 0.043, *p* = 0.020 and *p* = 0.011, respectively). No significant differences were observed between STZ, GFP + STZ, and GDNF + STZ groups (*p* > 0.05) (Fig. 5C). These results confirm that SHAM animals exhibited significantly lower microglial reactivity compared to all other groups.

## Discussion

This study describes the implementation of a bicistronic AAV9 vector designed to selectively overexpress GDNF in astrocytes. This approach enabled precise targeting of astrocytes in the hippocampus, providing new insights into the structural and functional effects of astrocytic GDNF overexpression in a neurodegenerative context.

Previous studies have explored GDNF overexpression in astrocytes using transgenic mouse models. For instance, transgenic mice overexpressing GDNF under the full GFAP promoter showed paracrine-mediated protection of motoneurons, preventing axotomy-induced cell death^38^. These animals also demonstrated enhanced survival of adult facial motoneurons following avulsion^39^. While these studies were pivotal, viral vectors have become the predominant tool for achieving localized GDNF expression. For example, a recombinant adenovirus expressing GDNF under the GFAP promoter was injected into the striatum of a Parkinson’s disease rat model, demonstrating neuroprotective effects^40^. In vitro studies have further corroborated the protective effects of astrocyte-derived GDNF, showing that conditioned media from GDNF-expressing astrocytes protects neuronal cultures from 6-OHDA toxicity^41^ and suppresses zymosan A-mediated microglial activation^42^. Moreover, GDNF has been implicated in astrocyte and astroglioma cell proliferation and migration, mediated through the GFRα1/RET/MAPK/pCREB/LOXL2 signaling axis, suggesting that its impact on glial cells may depend on the pathological context^43^.

The expression of TdTom from our vector confirmed precise astrocyte targeting, and GDNF expression was successfully achieved at the RNA and protein levels, as confirmed by RT-qPCR and immunohistochemistry in the hippocampus, respectively. Astrocytic GDNF overexpression significantly increased branching complexity and process length of astrocytes in both naïve and STZ-injected rats, as demonstrated by Sholl analysis. An interesting study showed that the GDNF-GFRα1 complex promotes dendritic growth and synaptic differentiation in hippocampal neurons^44^. Additionally, Bonafina and cols showed that GDNF regulates dendritic complexity and spine density in adult-born granule neurons via GFRα1, highlighting the role of astrocyte-derived factors in maintaining synaptic plasticity^45^. The observed enhancement of astrocytic branching complexity and process length in our model further underscores the versatile role of GDNF in promoting structural plasticity across neural cell types.

Previous work using lentiviral vectors to target astrocytes in the hippocampus of aged Fischer 344 rats^28^ and 3xTg-AD mice^29^ demonstrated improvements in the Morris water maze spatial memory task. Whereas aged rats and 3xTg-AD mice exhibit gradual, age-related memory impairments, the STZ induces a rapid and severe neuroinflammation. Our data partially align with those neurodegeneration models, as in our case GDNF overexpressed in astrocytes partially prevented memory deficit. Thus, GDNF + STZ animals exhibited a comparable performance to SHAM animals in locating the goal sector in the BM PT. However, the same group failed to show improvements in recognition memory. The discrepancy in the impact of GDNF therapy on spatial versus recognition memory may reflect differences in the neural circuits underlying these tasks. Spatial memory relies heavily on hippocampal integrity^46^, while recognition memory engages broader networks, including cortical and subcortical regions such as the medial prefrontal cortex, perirhinal cortex, and amygdala^47^. Since our approach targeted hippocampal astrocytes, the lack of improvement in recognition memory could be explained by the involvement of these other regions, where astrocytic GDNF overexpression did not take place. Moreover, the temporal dynamics of the tasks may contribute to the observed differences. Recognition memory in the NOR test was assessed as a short-term memory task (90 min after the training phase), whereas spatial memory in the BM PT involves a longer temporal window and repeated learning trials, engaging processes of memory formation and consolidation that may be differentially affected by GDNF treatment. Finally, the findings of a paper by Morrone and cols in TgF344-AD rats further emphasize the importance of regional contributions to cognitive outcomes, as rescue of certain cognitive functions depended on targeting specific processes and circuits^48^. These observations highlight the potential need for combinatorial therapeutic approaches addressing both hippocampal and extra-hippocampal regions to achieve comprehensive cognitive improvements in neurodegenerative conditions.

A key finding of our study is the lack of significant anti-inflammatory effects of astrocytic GDNF in the STZ-icv model. The GDNF + STZ group showed elevated levels of reactive microglia, comparable to the STZ and GFP + STZ groups, contrasting with the *in vitro* findings from Rocha and cols^42^, where astrocyte-derived GDNF inhibited microglial activation. This discrepancy likely stems from substantial differences between our in vivo model and their *in vitro* paradigm. The profoundly altered metabolic and inflammatory environment of the ICV-STZ model^8^ may overshadow the potential benefits of astrocyte-derived GDNF. In particular, STZ-induced insulin resistance in astrocytes reduces endogenous neurotrophic factor expression, including GDNF, and disrupts the IR/IRS-1/Akt signaling pathway^49,50^, which is part of the downstream cascade activated by GDNF through RET. In line with this, Konishi et al. (2014) demonstrated that neurons derived from AD brains exhibited deficient expression of GFRα1 and failed to respond to GDNF stimulation, highlighting that GDNF signaling may be intrinsically impaired in neurodegenerative conditions^51^. This disruption may impair the responsiveness to exogenous GDNF and alter astrocyte metabolism, promoting a glycolytic shift and increased lactate production. These changes could impair astrocyte-neuron metabolic coupling and, combined with altered insulin signaling, create a microenvironment less permissive for GDNF-mediated neuroprotection.

While our study focused on the structural and functional consequences of GDNF overexpression in astrocytes, we did not assess insulin-related signaling pathways such as Akt/PKB or GSK-3β, nor neuronal apoptosis markers such as cleaved caspase-3 or Bax/Bcl-2 ratio, which are often affected in this model^18,52^. Future studies should include these analyses to determine whether astrocyte-derived GDNF can modulate insulin and apoptotic signaling and promote neuronal survival in metabolically compromised and neurodegenerative conditions.

Numerous studies have demonstrated that microglial activation contributes to cognitive impairment in AD models, including the ICV-STZ paradigm. Early after STZ injection, reactive microgliosis and elevated proinflammatory cytokines such as TNF-α are detected in the hippocampus, coinciding with oxidative stress and synaptic dysfunction^52–54^. These events precede neuronal loss and appear to disrupt hippocampal neurogenesis and synaptic integrity, key mechanisms underlying memory impairment^52,54–56^. In our model, although cognitive performance improved following astrocytic GDNF overexpression, microglial reactivity remained unchanged. This suggests that the cognitive benefits observed were primarily mediated through astrocyte-neuron interactions rather than modulation of microglial activation. While STZ-induced metabolic alterations may interfere with astrocyte-neuron coupling, it is possible that GDNF exerts compensatory effects through alternative mechanisms—such as synaptic maintenance—that support neuronal function and enhance cognition independently of changes in microglial activation. Previous work has shown that astrocyte-derived factors can support synaptic plasticity independently of microglial state^55^, and our findings align with the idea that enhancing astrocytic neurotrophic support may suffice to improve cognitive function, even in the absence of changes in microglial activation. Moreover, while GDNF has been reported to reduce microglial activation in certain conditions, e.g., neuropathic pain model^57^, other studies found no such effects, depending on the disease model or delivery strategy^58^. Thus, the lack of microglial modulation in our study could reflect specific features of the ICV-STZ model.

Interestingly, previous work from our group demonstrated that IGF1 gene therapy reduced microglial reactivity and promoted hippocampal neurogenesis in ICV-STZ injected animals^35^, supporting its potent neuroprotective effects. As mentioned, in our study astrocytic GDNF failed to reduce microglial reactivity and partially improved spatial memory, therefore indicating that astrocyte-derived neurotrophic support can benefit cognition even in the absence of microglial modulation. Together, these observations reinforce the idea that multiple, complementary mechanisms—such as inflammation control and neural support—may be required to achieve broad and sustained cognitive benefits in neurodegenerative contexts.

Notably, Deng and cols demonstrated that GDNF treatment in astrocyte-Schwann cell co-cultures significantly reduced astrocyte hypertrophy and increased process length. Similar effects were observed *in vivo*, where GDNF reduced GFAP expression and enhanced axonal regeneration^59^. These results highlight the capacity of GDNF to modulate both the biochemical and morphological responses of astrocytes. Moreover, Bonafina and cols demonstrated functional improvements in behavioral pattern separation mediated by neuronal GDNF^45^. Taking all these results together, our findings highlight a limitation of GDNF in our neurodegeneration model. Although the neurotrophic factor prevented STZ-mediated astrocyte process length reduction, this was not sufficient to reduce neuroinflammation nor to preserve cognitive function at the NOR test. Persistent inflammation may negate the potential benefits of GDNF-induced astrocytic morphological changes. Furthermore, the observed lack of anti-inflammatory effects hints that the efficacy of GDNF may be context-dependent, influenced by the severity and duration of the inflammatory response. These findings underscore the need for further studies to delineate the specific pathways through which GDNF influences neuroprotection. A deeper understanding of these mechanisms is essential for developing therapeutic strategies that effectively modulate astrocytic function and mitigate neuroinflammation.

Finally, a limitation of this study is the exclusive use of male rats—a decision made to reduce variability associated with the estrous cycle and to maintain consistency with earlier studies. However, given the higher prevalence of AD in women and emerging evidence of sex-specific responses in preclinical models, future studies should include both sexes. In line with this, our group recently demonstrated that ICV-STZ induces differential cognitive and molecular outcomes in female rats depending on their ovarian status, highlighting the importance of considering sex as a biological variable in AD research^60^.

## Conclusion

This study introduces an AAV9 vector capable of selectively manipulating astrocytes, providing a powerful tool for investigating glial-specific contributions to brain function. Although GDNF overexpression enhanced astrocyte branching complexity and process length and partially improved hippocampal-dependent memory, it failed to reduce neuroinflammation in the ICV-STZ neurodegeneration model. These findings underscore the complexity of glial-neuronal interactions and suggest that morphological remodeling of astrocytes alone is insufficient for fully restoring cognitive function in the presence of neuroinflammation. This work contributes to GDNF research and paves the way for future studies aimed at refining astrocytic gene delivery strategies to achieve therapeutic benefits in neurodegenerative and neuroinflammatory diseases. Future research should focus on elucidating the signaling pathways activated by astrocytic GDNF and exploring combination strategies targeting both hippocampal and extra-hippocampal regions to achieve broader therapeutic benefits in neurodegenerative conditions.

## Electronic supplementary material

Below is the link to the electronic supplementary material.

Supplementary Material 1

Supplementary Material 2

Supplementary Material 3

## Data Availability

Data will be made available from the corresponding author (joaquin.pardo@med.lu.se) on reasonable request.

## Abbreviations

AAV: Adeno-associated virus
AD: Alzheimer’s disease
AT: Acquisition trial
BM: Barnes maze
GDNF: Glial cell line-derived neurotrophic factor
GFP: Green fluorescent protein
ICV: Intracerebroventricular
NOR: Novel object recognition
PT: Probe trial
SR: Stratum radiatum
STZ: Streptozotocin
TdToM: TdTomato
