# Supplementary figures and images for: GDNF overexpression in astrocytes enhances branching and partially preserves hippocampal function in an Alzheimer’s rat model
Source: Sci Rep. 2025 Jun 2;15:19284. doi: 10.1038/s41598-025-02881-4 (PMC12130339; doi:10.1038/s41598-025-02881-4)

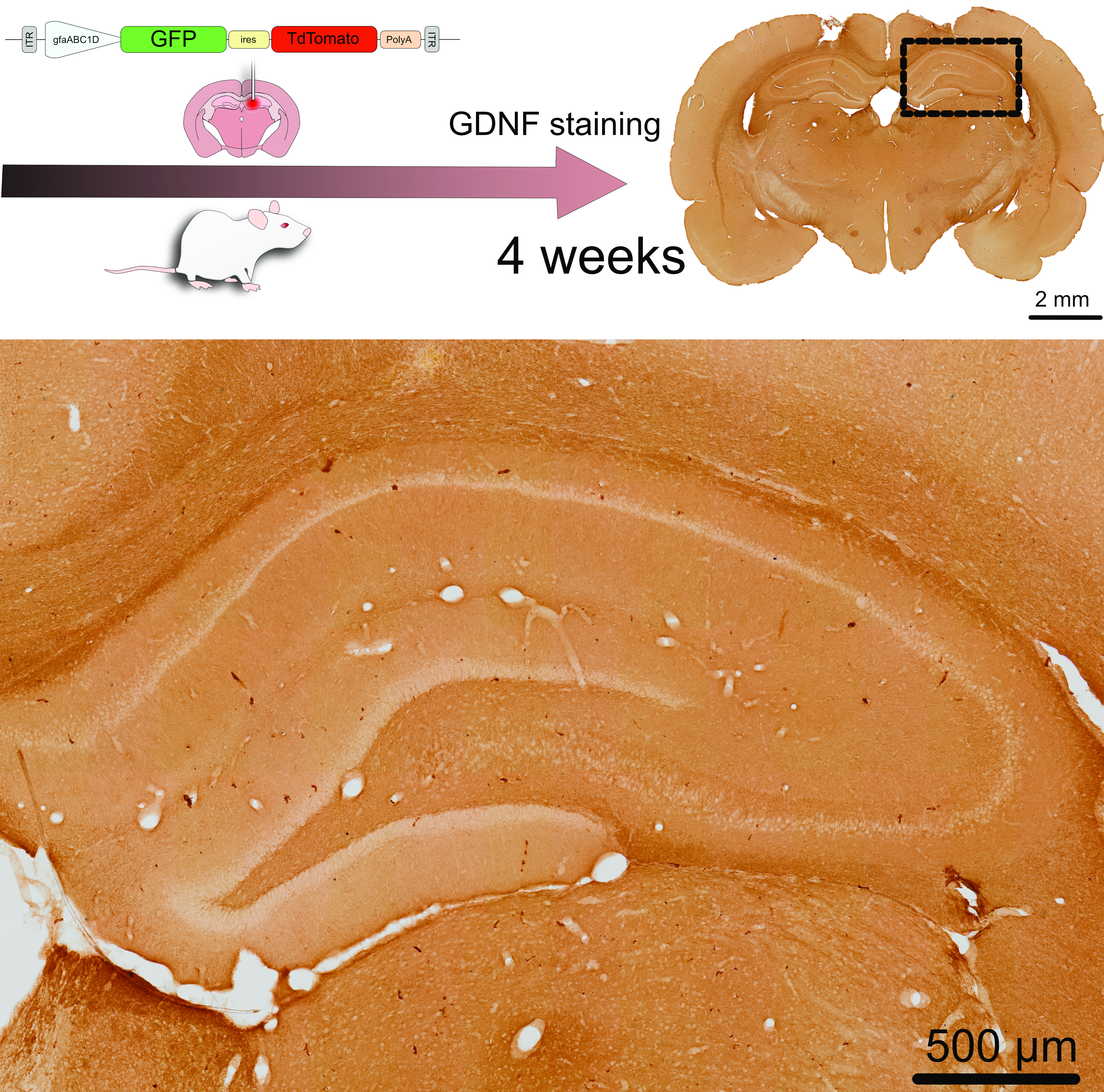

Supplement: Supplementary file 1 — Supplementary Material 1 [file 41598_2025_2881_MOESM1_ESM.tiff]

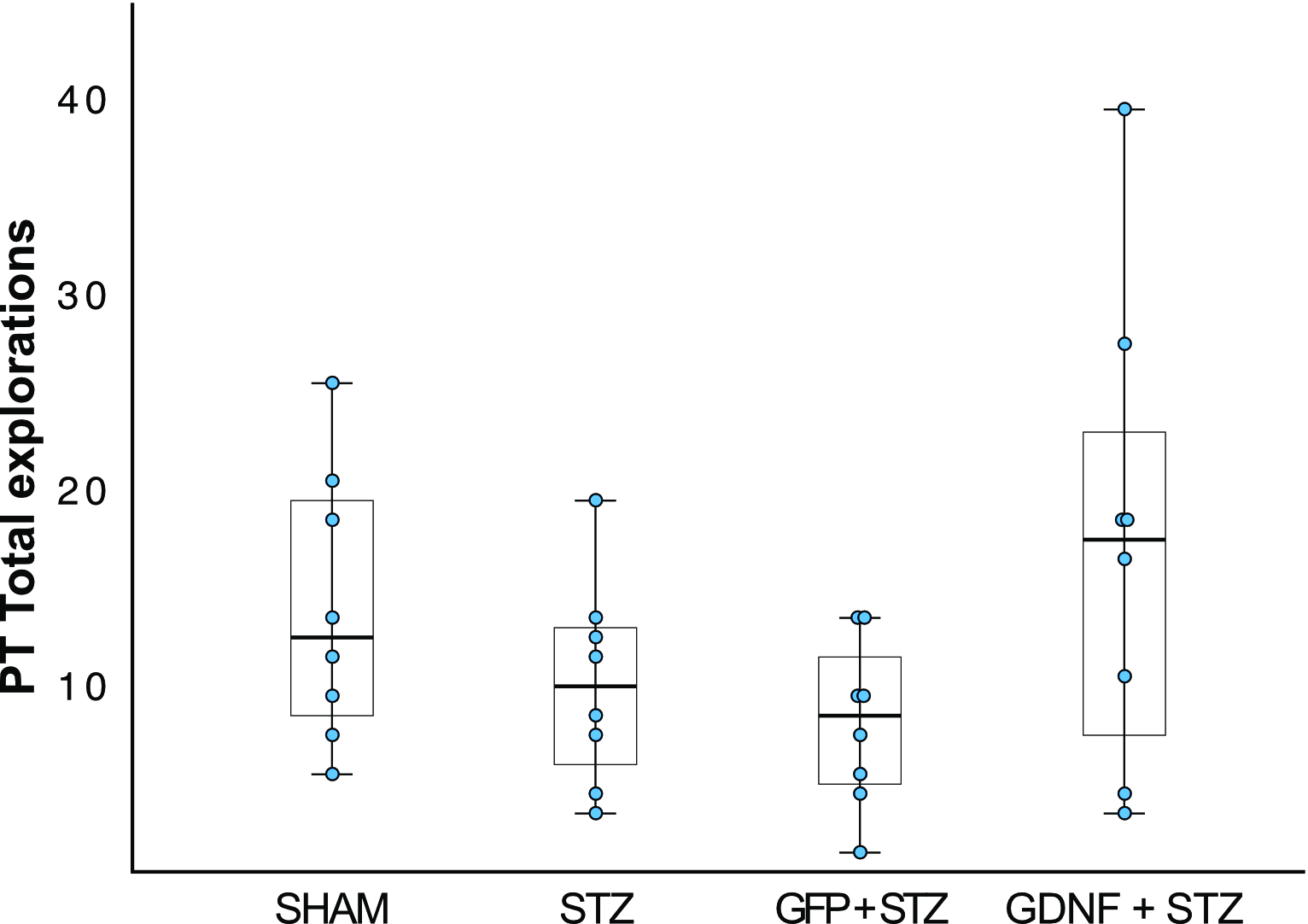

Supplement: Supplementary file 2 — Supplementary Material 2 [file 41598_2025_2881_MOESM2_ESM.tiff]
