# Supplementary material for: GDNF overexpression in astrocytes enhances branching and partially preserves hippocampal function in an Alzheimer’s rat model
Source: Sci Rep. 2025 Jun 2;15:19284. doi: 10.1038/s41598-025-02881-4 (PMC12130339; doi:10.1038/s41598-025-02881-4)
Supplement: Supplementary file 3 — Supplementary Material 3 [file 41598_2025_2881_MOESM3_ESM.docx]

**Supplementary figure 1. GDNF staining of an animal unilaterally injected with the GFP vector.** Experimental design and microphotographs showing that the GDNF antibody does not stain a section of an animal unilaterally injected with the mentioned control vector.

**Supplementary figure 2. Barnes maze total explorations.** Box plot with overlaid scatter plot showing total explorations in the BM PT.
